# Clearing the Brain’s Cobwebs: The Role of Autophagy in Neuroprotection

**DOI:** 10.2174/157015908784533897

**Published:** 2008-06

**Authors:** Blaise Bossy, Guy Perkins, Ella Bossy-Wetzel

**Affiliations:** 1 University of Central Florida, Burnett School of Biomedical Sciences, College of Medicine, 4000 Central Florida Blvd, Orlando, FL 32816, USA;; 2 National Center for Microscopy and Imaging Research, School of Medicine, University of California, San Diego, La Jolla, CA 92093, USA

**Keywords:** Autophagy, neurons, neurodegeneration, aggregates, mitochondria, cell death, apoptosis, survival.

## Abstract

Protein aggregates or inclusion bodies are common hallmarks of age-related neurodegenerative disorders. Why these aggregates form remains unclear. Equally debated is whether they are toxic, protective, or simple by-products. Increasing evidence, however, supports the notion that in general aggregates confer toxicity and disturb neuronal function by hampering axonal transport, synaptic integrity, transcriptional regulation, and mitochondrial function. Thus, neuroscientists in search of effective treatments to slow neural loss during neurodegeneration have long been interested in finding new ways to clear inclusion bodies. Intriguingly, two studies using conditional neuron-specific gene ablations of autophagy regulators in mice revealed that autophagy loss elicits inclusion body formation and a neurodegenerative cascade.Such studies indicate autophagy may be a built-in defense mechanism to clear the nervous system of inclusion bodies.This new finding has implications for our understanding of aging and neurodegeneration and the development of new therapies. First, we discuss the pathways underlying autophagy and its controversial role in cell death and survival regulation.We then discuss the physiological role of autophagy in the aging process of the nervous system. In the final portion of this review, we discuss the therapeutic promise of inducing autophagy and the potential side effects of such treatments.

## INTRODUCTION

**Fig. (1):**
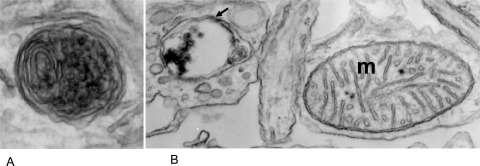
**A**. EM micrograph of an autophagosome. Note the typical double membrane wrap. **B**. EM micrograph of an autophagosome (arrow) next to a mitochondria (m).

Macroautophagy (here simply called autophagy) is a cellular housekeeping process that degrades and recycles long-lived proteins, large protein aggregates, and even entire organelles like mitochondria. The term autophagy is of Greek origin and translates literally as “the eating of oneself.” Autophagy is a dynamic process in which double membranes target and envelop cytoplasmic contents forming cytosolic vesicles, the autophagosomes (Fig. **1**). Autophagosomes then fuse with lysosomal vacuoles that contain acidic hydrolases and other degradative enzymes. After the release of lysosomal enzymes into the lumen of autophagosomes and the subsequent degradation of the contents, free amino acids and breakdown products are released into the cytoplasm and recycled for *de novo* biosynthesis.

Autophagy plays a physiological role in development, defense against pathogens, and adaptation to starvation. For example, amino acid release by autophagic degradation allows newborns to maintain a stable energy level for survival after birth [21]. Thus, it should come as no surprise that studies link autophagy dysfunction and pathophysiological situations to diseases like cancer, myopathy, and neurodegeneration. For example, a defect in autophagy causes Danon’s disease, which results in cardiomyopathy and mental retardation [27,38].

## MOLECULAR MECHANISM

Researchers are beginning to identify the molecular machinery responsible for autophagy in mammalian cells. Because autophagy is evolutionarily conserved, the discovery of autophagy related genes (Atg) in yeast laid the foundation for the identification of their mammalian orthologs. Although the picture remains incomplete, it is clear that a combination of kinases, phosphatases, guanosine triphosphatases (GTPases), and ubiquitin-like proteins direct the autophagy process.

**Fig. (2):**
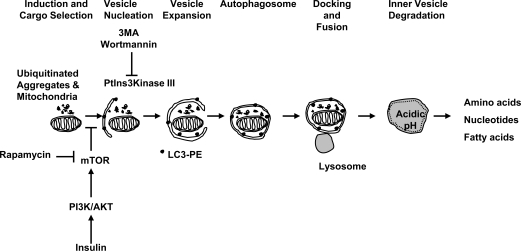
Schematic model of the molecular events regulating autophagy.

Autophagy consists of multiple steps (Fig. **2**) executed by specialized molecules. During the initiation phase, induced by starvation or loss of insulin signaling, the kinase target of rapamycin (TOR, in yeast), a nutrient sensor and negative regulator of autophagy, becomes inactivated. Rapamycin is an effective inhibitor of TOR, as well as of its mammalian ortholog (mTOR), and promotes autophagy (Fig. **2**). Upon deactivation of TOR, Atg13 becomes partially dephosphorylated and associates with Atg1, a serine/threonine kinase, which also binds to Atg17. Atg13 binding activates Atg1 and initiates autophagy [1,19].

The second phase of the autophagy process involves the selection and targeting of the cargo into vesicles (Fig. **2**). The cargo may consist of unfolded, ubiquitinated proteins too large for the proteasome to degrade, inclusion bodies, and injured mitochondria. The signals responsible for selecting the cargo for vesicle packaging are currently not clear. Equally unknown is the origin of the membranes that form the autosomal vesicle, but the endoplasmic reticulum (ER) is a candidate. At the molecular level, a class III phosphatidylinositol 3-kinase (PtdIns3Kinase) complex, which includes Atg6 (called Beclin-1 in mammals), mediates the vesicle nucleation. 3-Methyladenine and Wortmannin can inhibit PtdIns3Kinases and prevent autophagy.

The third phase in autophagy involves vesicle expansion (Fig. **2**), mediated by a series of steps that involve two ubiquitin-like conjugation systems (reviewed in [10,13]). In one step, Atg5 and Atg12 become covalently bound after sequential interaction with Atg7 and Atg10 (E1-like and E2-like ligase proteins respectively). In a second conjugation pathway, Atg8, also called LC3 in mammals, undergoes a series of modifications. First, Atg 4 cleaves Atg8. Next, Atg7 activates Atg8, which then binds to Atg3. Finally, Atg8 is conjugated to phosphatidyl-ethanolamine (PE) [17].

In the last step, the autophagosomes bind to and fuse with lysosomes for bulk degradation of their contents (Fig. **2**). After degradation, free amino acids, nucleotides, fatty acids and breakdown products are released into the cytosol and recycled.

## AUTOPHAGY: CELL SURVIVAL OR CELL DEATH?

The exact role of autophagy in the nervous system is a matter of controversy. One view is that autophagy represents a compensatory, neuroprotective mechanism to clear toxic accumulation of misfolded proteins or defective mitochondria, thereby slowing synaptic damage and increasing neuronal survival. Along this line, studies have found that mTOR, an inhibitor of autophagy, becomes trapped in inclusion bodies, promoting autophagy induction and clearance of toxic aggregates [30]. Additionally, rapamycin, an autophagy inducer, protects against the cytotoxic effects of different aggregate-prone proteins including polyglutamine containing proteins and mutant Tau [3].

The opposing view derives from the observation that autophagosomes accumulate during a non-apoptotic form of cell death known as type II cell death [4]or autophagic cell death [12,41]. Although autophagosomes are present in both healthy and dying cells, they are frequently found at increased levels in dying neurons. This observation leads to the notion that autophagy, or at least part of its machinery, might play a role in a cell death pathway [13]. Alternatively, proponents of the cell survival role of autophagy hypothesize that increased levels of autophagosomes found in dying or dead cells result from a failed survival attempt by the cell.

While the causal relationship between autophagy level and cell death is unclear, several studies have linked increased rates of autophagy with different characteristics of cell death and degeneration. For example, studies have linked elevated levels of autophagy with increased amyloid precursor protein processing and amyloid β peptide generation associated with neurodegenerative diseases such as Alzheimer’s disease. In addition, researchers detected Aβ peptide production within autophagosomes [43]. Furthermore, Lurcher mice, whose mutant phenotype results from an aggregate-prone protein, display autophagy associated with neuronal cell death [44].

Further complicating the picture is the considerable overlap between the autophagic and apoptotic machinery. For example, Bcl-2 functions normally as an anti-apoptotic protein, but is anti-autophagic when it is ER-located and inhibits the protein Beclin-1 (Atg6) during autophagosome nucleation [9,16,28]. Another example of the autophagic and apoptotic overlap involves embryonic fibroblasts. Embryonic fibroblasts treated with staurosporine undergo apoptosis. However, Bax/Bak knockout embryonic fibroblasts, which are resistant to apoptosis, undergo Beclin-1 dependent autophagic cell death in response to staurosporine [37].

Atg5 is another link between autophagy and apoptosis. Atg5 expression induces autophagy linked to cell death by interacting with the Fas-associated death domain protein (FADD) [29]. In addition, proteolytic cleavage at the N-terminus by the calcium dependant protease calpain targets Atg5 to mitochondria and subsequent binding to Bcl-xL mediates apoptotic cell death [40]. Expression of a truncated form (1-193) of Atg5 is sufficient to evoke apoptotic cell death in the absence of autophagy, indicating that the autophagic and apoptotic functions of Atg5 are independent of each other [40].

Ultimately, the cell survival and death functions of autophagy might not be mutually exclusive and the balance between the two functions is likely critical in determining cellular fate. For example, Atg5 overexpression in HeLa cells promotes both autophagy and cell death [29]. In addition, studies have shown that several autophagy genes, including Atg5, Atg8, and Beclin-1 in mammalian cells, are involved in autophagic cell death [29,37,41]. Stressed cells defective for the apoptotic pathway can undergo autophagic cell death, which anti-autophagy inhibitors such as 3-MA or Wortmannin can inhibit [41]. While the pan-caspase inhibitor, zVAD, blocks apoptosis, L929 cells die by autophagic cell death. Similarly, caspase-8 inhibition by RNAi in the presence of zVAD leads to enhanced autophagic cell death in the same cell line [41]. Finally, stressing apoptosis-deficient cells and denying autophagy induces necrotic cell death [18].

In sum, these observations led to the idea that autophagic cell death is a “fail-safe”, non-apoptotic cell death mechanism [41]. An important implication of this cross-talk between cell death pathways is that the efficient blockage of cell death might require multiple inhibitors.

As we will discuss further in the following sections, autophagy is basically a survival mechanism. In some situations of cellular stress, however, cells can use parts of the autophagic machinery to generate cell death when apoptosis is inhibited or deficient. For example, it has been proposed that a critical step leading autophagy to a lethal outcome in L929 fibroblast cell lines is the degradation of catalase and the generation of reactive oxygen species (ROS) [42]. Caspase-8 inhibition in L929 cells activates the receptor interacting protein (RIP), Jun kinase (JNK), and autophagy with selective catalase degradation and an accumulation of ROS, ultimately leading to cell death. In contrast, starvation induced autophagy in the same cell line does not lead to catalase degradation, but instead promotes cell survival, indicating that different autophagic stimulations can lead to different outcomes [42]. Finally, apoptosis and autophagic cell death are not mutually exclusive and may occur in the same cell at the same time [11,13,22].

## ROLE OF AUTOPHAGY IN NEURONS

To understand the relationship between neuronal pathology and autophagy, two independent groups generated conditional Atg7 and Atg5 null mice, respectively [15,20]. Interestingly, they found that neuron-specific Atg7 and Atg5 gene deletion in mice evokes progressive motor deficits, neurodegeneration, and inclusion body formation without corresponding defects in proteasome function, indicating that autophagy loss alone sets off a neurodegenerative cascade.

Komatsu and his colleagues generated nervous system specific Atg7 conditional knockout mice. Atg7 is an E1-like enzyme required for autophagic vacuole formation. Brain-specific Atg7 deficient mice exhibit reduced survival, up to 4 weeks, and reduced body size. Most importantly, Atg7 knockout mice reveal limb-clasping, abnormal reflexes, reduced movement coordination, and tremors indicating severe neurological defects. Neuronal loss was evident in the cerebral and cerebellar cortex. Specifically, pyramidal neurons were missing and signs of gliosis, which is a diagnostic indicator of neurodegenerative events, appeared. As the Atg7 knockout mice aged, there was an increase in ubiquitin-containing inclusion bodies in their brains. Inclusion body formation was clearly due to a decrease in autophagy, because proteasome function remained normal. Similarly, Hara and colleagues reported a neurodegenerative phenotype for brain-specific Atg5 null mice [15]. These mice showed motor deficits, Purkinje cell loss, and inclusion bodies. Taken together, these findings suggest that autophagy plays a pivotal role in preventing inclusion body accumulation and neurodegeneration, providing a neuroprotective effect.

## AUTOPHAGY AND AGING

Studies indicate that autophagy may increase longevity [2]. For example, caloric restriction, which has proven to increase life span in rodents, leads to dramatic induction of autophagy [26]. Genetic studies in the nematode, *Caenorhabditis elegans*, further support the view that autophagy may slow aging. Stressors like limited food, high population density, heat, or exposure to the “dauer” hormone lead to reversible arrest in the third larval stage of the nematode [14]. This phase, the “dauer diapause”, is an adaptive stage that allows the organism to survive unfavorable and harsh environmental conditions. Rates of autophagy dramatically increase during dauer diapause [25]. However, ablation of the nematode ortholog of the autophagy gene beclin-1 abolishes dauer survival and reduces life span. On the other hand, loss-of-function mutations in autophagy inhibitors such as Akt, TOR, and PtdIns3-kinase, all extend the life span of nematodes [14,39]. These observations are consistent with the view that inhibition of autophagy repressors may extend life span.

Another potential mechanism of autophagy anti-aging effects is the removal of damaged mitochondria, which leak free radicals. Studies have implicated reactive oxygen species in accelerating aging [5]. Various cellular stress signals can lead to mitochondrial outer membrane permeability. Interestingly, mitochondria with increased mitochondrial membrane permeability are selectively targeted into autophagosomes [23]. Thus autophagy of damaged mitochondria might reduce oxidative damage. Autophagy of mitochondria can also have other clinical implications. For example, studies have linked lysosomal accumulation of subunit 9 of mitochondrial ATPase owing to incomplete degradation of mitochondria with neuronal ceroid lipofuscinosis and neurodegenerative disease [7].

With age, deactivation of autophagy can occur, which in turn triggers age-related accumulation of inclusion bodies and consequent neurodegeneration. Investigations of post-mortem brain tissue of AD and PD patients have revealed elevated autophagosome levels and decreased levels of Beclin-1 [36]. The aging pigment lipofuscin accumulates in lysosomes and the subsequent accumulation of indigestible lysosomal contents inhibits autophagy [5]. Additionally, recent reports indicate that mutant Huntingtin (mtHtt) protein recruits Beclin-1, which impairs autophagy, setting off a vicious cycle of protein accumulation [36]. Similarly, dynein mutations cause ALS-like symptoms in mice. Dynein is a motor protein important for autophagosome-lysosome fusion. Decreased dynein function results in autophagy and aggregate clearance defects [31]. Whether motor proteins and microtubule network defects play a role in limiting autophagy induction in the aging nervous system remains a topic for future investigation.

To summarize, numerous studies point to the anti-aging properties of autophagy. Possible mechanisms of anti-aging activity include clearance of protein aggregates and the removal of damaged mitochondria. Research indicating that decreased rates of autophagy with age lead to inclusion body accumulation and neurodegeneration further solidifies the view that autophagy is an important cell survival mechanism.

## THERAPEUTIC STRATEGIES

The knowledge that autophagy can clear toxic aggregates offers new opportunities for the development of drugs to combat progressive, age-related neurodegenerative disorders. Rapamycin, a drug currently used in humans to inhibit renal transplant rejection, is an early candidate for autophagy induction therapy. Rapamycin alleviates neurotoxicity by clearing toxic, aggregate-prone proteins such as mtHtt or mutant Tau in animal models [3]. Additionally, it may offer added protective effects by removing injured mitochondria, which can leak ROS and cytochrome c. Researchers have also tested in clinical trials the ability of rapamycin to alleviate gliomas in combination with anti-tumor drugs [8,33]. While physicians already use rapamycin clinically, its effect on the human nervous system remains unknown. Because mTOR signaling affects multiple pathways in addition to autophagy, rapamycin, although specific for mTOR, may have potential side effects. Among them is impaired wound healing and immune suppression. Nevertheless, prolonged use of rapamycin in cancer patients has not shown evidence of autophagy related side effects [34].

Although not yet demonstrated, long-term stimulation of autophagy might have additional risks. One concern is that elevated autophagy may lead to deleterious depletion of healthy mitochondria. Neurons are particularly sensitive to bio-energetic failure. In fact, mitochondrial dysfunction is an early event in the pathogenesis in many neurodegenerative disorders [24]. However, some respiratory complexes can be lowered 25 to 80% before brain mitochondria show evidence of lower rates of respiration or ATP production [6]. In addition, the lower steady state level of mitochondrial mass resulting from enhanced autophagy *in vitro* does not result in complete mitochondria depletion, but rather decreases the amount by about 50% [32].

A potentially interesting approach to autophagy induction therapy is the use of a cocktail of autophagy inducers. For example, recent studies show that trehalose, a disaccharide sugar, enhances rates of autophagy independently of mTOR [35]. In addition, trehalose and rapamycin have an additive effect on the clearance of aggregate-prone proteins such as huntingtin or α-synuclein mutant proteins. If we can establish that autophagy plays a beneficial role in acute brain injuries such as stroke, short-term treatment with autophagy inducers in combination with other neuroprotectants might prove useful in increasing neuronal recovery after brain injury.

## FUTURE DIRECTIONS

Several fundamental questions remain. What is the cause of the selective vulnerability of certain neurons to autophagy loss? Reciprocally, what determines the tissue and cell specificity of autophagy induction? What are the molecular sensors responsible for the initiation of autophagy? What are the molecular signals that target damaged organelles and aggregates to autophagosomes? It will also be important to gain further insight into the cross-talk between autophagy and apoptosis to shed more light on the delicate balance between survival and cell death. Finally, we have yet to demonstrate the therapeutic benefits of autophagy, notably the alleviation or delay of neurodegeneration, in humans. However, data from animal models and *in vitro* systems justify prudent optimism.
